# Transmembrane protein 88 suppresses hepatocellular carcinoma progression and serves as a novel prognostic factor

**DOI:** 10.3389/fonc.2023.1148498

**Published:** 2023-04-06

**Authors:** Lin Cai, Yu Du, Kai Song, Peng Peng, Fei Han

**Affiliations:** ^1^ School of Food and Drug, Xuzhou Polytechnic College of Bioengineering, Xuzhou, China; ^2^ Department of Traditional Chinese Medicine, Xuzhou Kuangshan Hospital, Xuzhou, China; ^3^ Department of General Surgery, Xuzhou Kuangshan Hospital, Xuzhou, China

**Keywords:** transmembrane protein 88, hepatocellular carcinoma, proliferation, xenografts, prognosis

## Abstract

**Background:**

Transmembrane protein 88 (TMEM88) is known to be involved in the canonical Wnt signaling pathway and is implicated in several malignancies. However, the expression, function, and prognostic significance of TMEM88 in hepatocellular carcinoma (HCC) remain unclear.

**Methods:**

In this study, we analyzed mRNA levels of TMEM88 in HCC specimens from the TCGA dataset (n=374) to explore the correlation between TMEM88 and HCC. We also overexpressed TMEM88 in the Huh7 human HCC cell line to investigate its tumor-related role in HCC. Additionally, we conducted *in vivo* experiments using a mouse model to further validate the critical function of TMEM88 in modulating HCC growth.

**Results:**

Our results showed that TMEM88 is negatively correlated with the T stage, TNM stage, and pathological grade of HCC. Higher levels of TMEM88 can help predict better overall survival of HCC in both univariate and multivariate analyses. Similarly, higher TMEM88 is a novel prognostic factor for better disease-specific survival of HCC. Overexpression of TMEM88 in Huh7 cells led to a decreased cell proliferation capacity. Xenograft experiments in a mouse model showed that TMEM88 overexpression can remarkably suppress HCC progression.

**Conclusions:**

Transmembrane protein 88 suppresses HCC growth both *in vitro* and *in vivo*, which can act as a potential prognostic factor with clinical application potential.

## Introduction

Hepatocellular carcinoma (HCC) represents a common malignancy with poor prognosis, ranking the fourth leading cause of cancer-related mortality worldwide (1). Predominant HCC risk factors include hepatitis virus infection, alcohol liver diseases, steatohepatitis, etc (2). Although for HCC patients diagnosed at early-stage, tumor resection and transplantation represent the curative therapies, a large amount of HCC patients are diagnosed at late stages with poor prognoses (3). In general, the five-year survival rate of HCC is less than 20% (4). Further exploration of the disease mechanisms and identification of prognostic biomarkers are critical for HCC treatment.

Transmembrane protein 88 (TMEM88) is a member of the TMEM protein family and was first identified in 2010 by Lee HJ and colleagues (5). It was initially recognized as a disheveled-binding protein, with the authors confirming that the PDZ domain of disheveled binds to the C-terminal domain of TMEM88 through various methods. Interestingly, the authors found that silencing TMEM88 led to enhanced Wnt/β-catenin signaling, suggesting a suppressing role of TMEM88 on this signaling pathway. Microarray analysis also revealed significant correlations between TMEM88 and genes in the Wnt/β-catenin pathway. Further investigation showed that TMEM88 enhances cardiomyocyte differentiation and suppresses endothelial differentiation by inhibiting the Wnt/β-catenin signaling pathway, highlighting the crosstalk between TMEM88 and Wnt signaling (6). Furthermore, TMEM88 participates in the inflammatory responses. For example, Tao et al. reported that TMEM88 promotes TNF-α-induced secretion of inflammatory factors in human hepatic stellate cells (7). Their group later reported that this regulation can be negatively regulated by miR-708, which can directly target TMEM88 3’-UTR regions (8). Similarly, different groups found that TMEM88 play multiple roles in liver diseases, including liver fibrosis (9), alcoholic liver disease (10), as well as non-alcoholic fatty liver disease (11).

Nevertheless, whether TMEM88 play potential roles in HCC remains unclear. Here in the current research, we identified that TMEM88 was negatively correlated with T stage, TNM stage, pathological grade, and plasma Alpha-fetoprotein (AFP) level. In addition, we confirmed the prognostic role of TMEM88 in HCC through statistical analyses. Tumor-related effects of TMEM88 in HCC were further validated through cellular and mice experiments.

## Methods

### Pan-cancer analysis

Expression levels of TMEM88 within normal tissues and cancer tissues (TCGA samples) were compared by Gene Expression Profiling Interactive Analysis (GEPIA) database (http://gepia.cancer-pku.cn/).

### Correlation evaluation of immune infiltration of TMEM88 in HCC

Here we utilized the TIMER algorithm (http://timer.cistrome.org/) to evaluate the relationship between TMEM88 in HCC and tumor immune infiltrations (B cells, T cells, T cells, TAM (Tumor-associated macrophage), M1 macrophages (M1), M2 macrophages (M2), Monocyte, Neutrophils, and Natural killer Cell, Dendritic cell).

### Cell culture and transfection

Huh7 was purchased from ATCC and cultured in RPMI 1640 medium supplemented with 10% fetal bovine serum (FBS), penicillin (100IU/mL) and 100mg/ml streptomycin.

The plasmids containing TMEM88 coding gene and control plasmids were established by GenePharma (Shanghai, China). Overexpression was achieved by transfection using the Lipo3000 Reagent (12, 13).

### Cell counting kit 8 experiments

Cellular viability was analyzed *via* CCK-8 kit. Briefly, after transfection and cultured for 24 hours, Huh7 cells were planted into the 96-well plates at a density of 3000 cells/well and incubated for 4 days. On each designated time point, each well was added with 10 μL of CCK-8 reagent, followed by 2 h incubation in the incubator. Finally, measurement of absorbance at 450 nm was conducted using a microplate reader.

### Mice xenograft assay

The protocol of animal experiments was approved and supervised by the Ethics Committee of Xuzhou Polytechnic College of Bioengineering. BALB/c nude mice (5 weeks old) were housed at 20−22°C and 55 ± 10% humidity with 12 h day/night cycles and were free to food and water. Tumor xenograft model was established by subcutaneous injection of transfected Huh7 cells *via* subcutaneous injection. The volume (V) of tumor was evaluated by the tumor width (W) and length (L) and calculated according the formula: V= (W × W × L)/2. One month after subcutaneous injection, mice were euthanatized and satisficed to isolate subcutaneous tumors. After tumor resection, the tumor weight was recorded and compared.

### Statistics

Data statistical analysis and graphing were performed using SPSS22.0 software and GraphPad Prism 6.0 software. Statistical differences were evaluated through Student’s t-test or One-way ANOVA test. Univariate and multivariate survival analyses were conducted for prognostic evaluation. Two sided P value less than 0.05 was considered statistical significance (14).

## Results

### TMEM88 was decreased in most tumor types comparing to normal tissues

**Figure 1:**
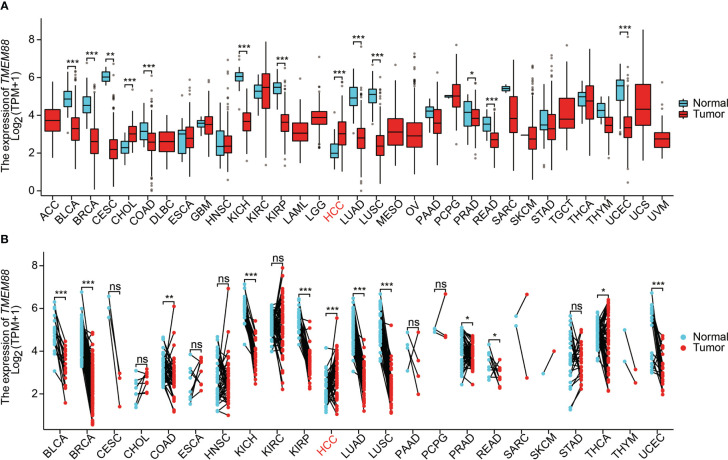
TMEM88 mRNA expression in different cancers. **(A)** Boxplot indicates the mRNA level of TMEM88 in normal tissues and tumor samples derived from different tumor types. Data was analyzed by Wilcoxon signed rank test. **(B)** The mRNA level of TMEM88 in specific tumor samples and their adjacent matched nontumorous tissues were compared. Each dot represents a sample specimen. Data was analyzed by Wilcoxon rank sum test. *P<0.05, **P<0.01,***P<0.001, ns, not significant.

To explore the possible tumor-related role of TMEM88, we firstly used in silico strategies to compare the mRNA level of TMEM88 in various tumors in TCGA datasets. As a result, comparing with normal tissues, TMEM88 was significantly decreased in bladder cancer, breast cancer, cervical cancer, colon cancer, rectum cancer, kidney chromophobe cancer, kidney renal papillary cell carcinoma, NSCLC, prostate cancer, and uterine corpus endometrial carcinoma (**Figure 1A**). Interestingly, the non-paired data showed that TMEM88 expressed higher in cholangiocarcinoma and HCC compared to nontumorous specimens (**Figure 1A**). Therefore, we further compared the differences in tumors and their paired nontumorous tissues (**Figure 1B**). Among the analyzed tumor types, higher TMEM88 was only observed in HCC comparing with its paired liver tissues. In contrast, lower TMEME88 or no statistical significance was observed in other tumor types (**Figure 1B**).

### TMEM88 was lower in HCC tissues that possessing more malignant phenotypes

The expression data of TMEM88 thus attracted us to further investigate whether it played any distinct roles in HCC. **Table 1** exhibited the basic information of the enrolled TCGA cohort. Briefly, there were 121 females and 253 males. Among them, 177 patients were diagnosed at 60 years old or younger ages, while the other 196 patients were elder at the time of diagnosis. Up to 65 patietns were characterized with serum AFP higher than 400 ng/ml. There were 55 patients with histologically grade G1, 178 pateints with G2, 124 patients with G3, and 12 patients with G4. According to the T stage, 183 patients were staged as T1, 95 patients as T2, 93 patients as T3-4. Vascular invasion was identified in 110 patients and showed negative invasion in 209 patients. Till the end of follow up, 130 patients died and 79 of them was classified as HCC-related death.

**Table 1:** Correlations between TMEM88 level with HCC characteristics.

Characteristics	Low expression of TMEM88	High expression of TMEM88	P value
n	187	187	
Gender, n (%)			0.320
Female	65 (17.4%)	56 (15%)	
Male	122 (32.6%)	131 (35%)	
Age, n (%)			0.957
≤ 60	88 (23.6%)	89 (23.9%)	
> 60	98 (26.3%)	98 (26.3%)	
Race, n (%)			0.617
Asian	84 (23.2%)	76 (21%)	
Black/African American	10 (2.8%)	7 (1.9%)	
White	90 (24.9%)	95 (26.2%)	
AFP(ng/ml), n (%)			0.332
≤ 400	101 (36.1%)	114 (40.7%)	
> 400	35 (12.5%)	30 (10.7%)	
Histologic grade, n (%)			0.015*
G1	22 (6%)	33 (8.9%)	
G2	81 (22%)	97 (26.3%)	
G3	73 (19.8%)	51 (13.8%)	
G4	9 (2.4%)	3 (0.8%)	
Pathologic T stage, n (%)			0.005**
T1	77 (20.8%)	106 (28.6%)	
T2	53 (14.3%)	42 (11.3%)	
T3&T4	57 (15.4%)	36 (9.7%)	
Pathologic stage, n (%)			0.004**
Stage I	72 (20.6%)	101 (28.9%)	
Stage II	48 (13.7%)	39 (11.1%)	
Stage III& IV	56 (16%)	34 (9.7%)	
Vascular invasion, n (%)			0.014*
No	89 (28%)	119 (37.4%)	
Yes	63 (19.8%)	47 (14.8%)	
OS event, n (%)			0.385
Alive	118 (31.6%)	126 (33.7%)	
Dead	69 (18.4%)	61 (16.3%)	
DSS event, n (%)			0.374
No	140 (38.3%)	147 (40.2%)	
Yes	43 (11.7%)	36 (9.8%)	

*P<0.05, **P<0.01.

**Figure 2:**
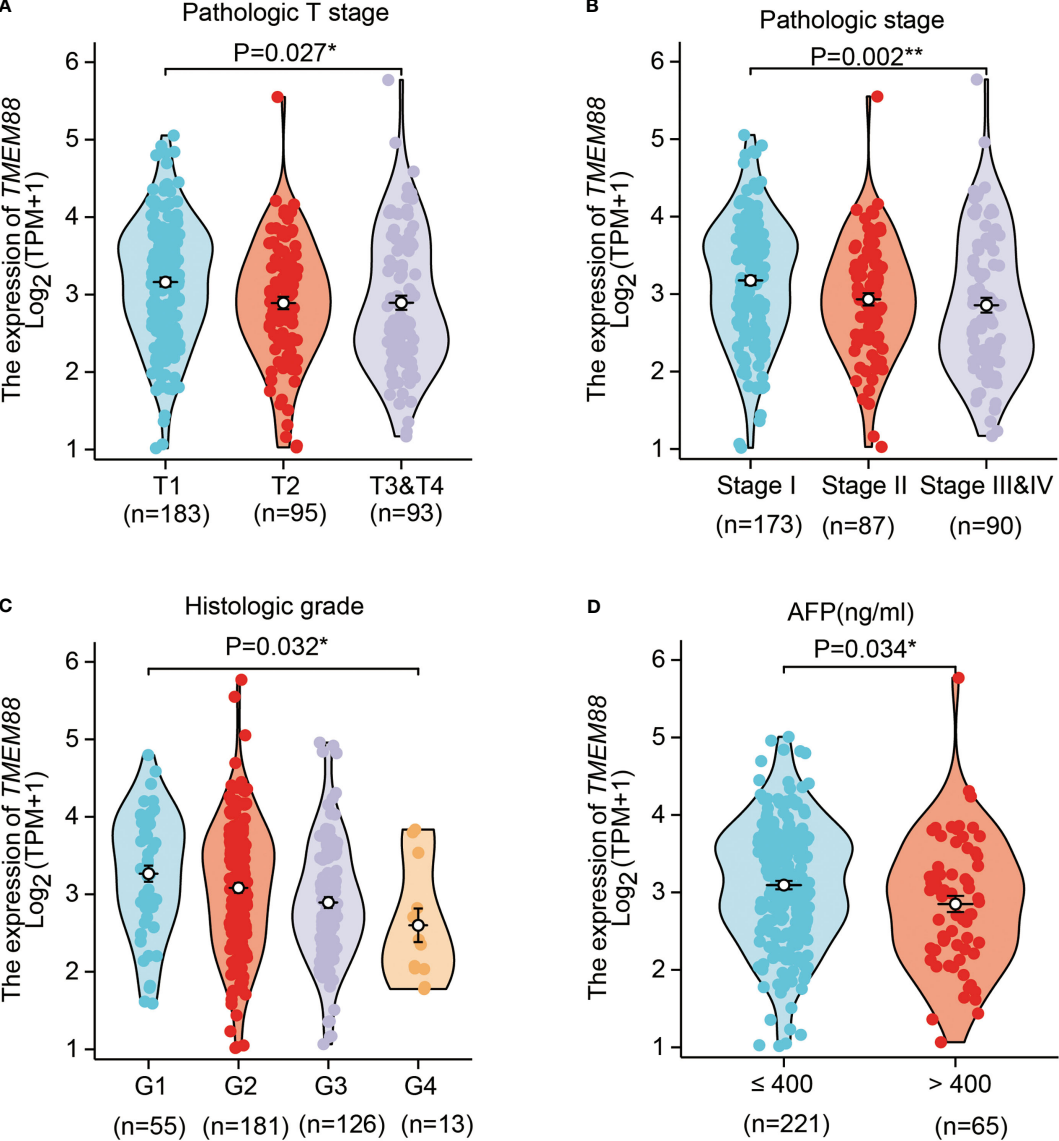
TMEM88 mRNA level was correlated with certain HCC characteristics. The correlations between TMEM88 mRNA levels with HCC T stage **(A)**, TNM stage **(B)**, histological grade **(C)**, and patients serum AFP level **(D)** were analyzed, respectively. Data was analyzed by Student’s t-test between each two groups. *P<0.05, **P<0.01.

By comparing its mRNA levels with Chi-square test, we found that TMEM88 showed significant different levels in different patient subgroups (**Table 1**). For example, patients with higher T stages showed lower TMEM88 levels in the HCC tissues (**Figure 2A**, P=0.027). Consistently, higher TNM stage was correlated with lower TMEM88 mRNA level (**Figure 2B**, P=0.003). Similar results were observed regarding the pathological differentiation stage on that HCCs with poorer stages exhibited lower TMEM88 mRNA level (**Figure 2C**, P=0.048). Of note, patients with higher serum AFP levels, which was well-acknowledged to possess worse prognosis, showed lower TMEM88 mRNA level (**Figure 2D**, P=0.034). The negative correlations between TMEM88 and malignant characteristics of HCC indicated that TMEM88 may be correlated with HCC prognosis.

### Lower TMEM88 was correlated with worse HCC prognosis

**Figure 3:**
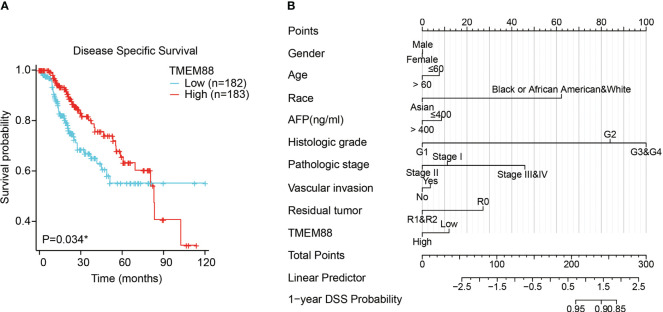
Lower TMEM88 predicted worse disease-specific survival of HCC. **(A)** Kaplan-Meier survival analysis and log-rank test showed that HCC patients with lower TMEM88 mRNA level exhibited worse disease-specific survival. **(B)** A prognostic predicting nomogram was established according to the multivariate analysis data to help predict 1-year disease-specific survival probability of HCC patients. *P<0.05.

Univariate survival analysis using either log-rank test (**Figure 3A**, P=0.034) or Cox hazard regression test (**Table 2**, P=0.035) both showed that HCC patients with lower TMEM88 showed worse disease-specific survival (DSS). HCC patients with advanced TNM stage also exhibited worse DSS [**Table 1**, hazard ratio (HR)=4.43, P<0.001]. However, multivariate analysis regarding DSS identified TNM stage as the only independent prognostic factor (**Table 2**, HR=4.21, P<0.001). Although patients with higher TMEM88 level showed a lower DSS risk (HR=0.62), the difference was not statistically significant (**Table 2**, P=0.062). Nevertheless, based on the multivariate data analysis, we established a nomogram model to help predict 1-year DSS of HCC patients (**Figure 3B**).

**Table 2:** Overall survival analyses.

Characteristics	Total (N)	Univariate analysis		Multivariate analysis	
		Hazard ratio (95% CI)	P value	Hazard ratio (95% CI)	P value
Gender	365		0.377		
Female	118	Reference			
Male	247	0.813 (0.516 - 1.281)	0.373		
Age	365		0.459		
≤ 60	174	Reference			
> 60	191	0.846 (0.543 - 1.317)	0.458		
Race	353		0.184		
Asian	156	Reference			
Black/African American	17	0.938 (0.222 - 3.964)	0.930		
White	180	1.539 (0.948 - 2.499)	0.081		
AFP(ng/ml)	275		0.665		
≤ 400	214	Reference			
> 400	61	0.867 (0.450 - 1.668)	0.668		
Histologic grade	360		0.839		
G1	55	Reference			
G2	172	1.177 (0.599 - 2.314)	0.636		
G3&G4	133	1.228 (0.613 - 2.462)	0.562		
Pathologic stage	341		<0.001***		
Stage I	170	Reference		Reference	
Stage II	84	1.560 (0.776 - 3.140)	0.212	1.460 (0.723 - 2.948)	0.291
Stage III& IV	87	4.434 (2.538 - 7.746)	<0.001***	4.210 (2.401 - 7.382)	<0.001***
Vascular invasion	309		0.425		
No	204	Reference			
Yes	105	1.277 (0.707 - 2.306)	0.418		
Residual tumor	337		0.258		
R0	320	Reference			
R1&R2	17	1.678 (0.728 - 3.870)	0.224		
TMEM88	365		0.035*		
Low	182	Reference		Reference	
High	183	0.620 (0.397 - 0.967)	0.035*	0.623 (0.380 - 1.023)	0.062

*P<0.05, ***P<0.001.

**Figure 4:**
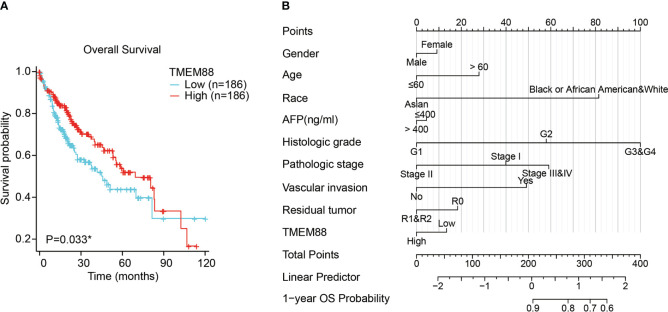
Lower TMEM88 predicted worse overall survival of HCC. **(A)** Kaplan-Meier survival analysis and log-rank test showed that HCC patients with lower TMEM88 mRNA level exhibited worse overall survival. **(B)** A prognostic predicting nomogram was established according to the multivariate analysis data to help predict 1-year overall survival probability of HCC patients *P<0.05.

Consistent with the DSS, overall survival (OS) data analyses also reflected a significant prognostic role of TMEM88 in HCC (**Table 3**). As shown in **Figure 4A**, lower TMEM88 predicted a worse OS of HCC (P=0.033). Moreover, multivariate Cox hazard regression analysis revealed that patients with higher TMEM88 had a lower hazard ratio (HR=0.074). However, no statistically significant difference was identified (**Table 3**, P=0.111). Nomogram to predict 1-year OS of HCC patients was shown in **Figure 4B**.

**Table 3:** Overall survival analyses.

Characteristics	Total (N)	Univariate analysis		Multivariate analysis	
		Hazard ratio (95% CI)	P value	Hazard ratio (95% CI)	P value
Gender	373		0.204		
Female	121	Reference			
Male	252	0.793 (0.557 - 1.130)	0.200		
Age	373		0.293		
≤ 60	177	Reference			
> 60	196	1.205 (0.850 - 1.708)	0.295		
Race	361		0.268		
Asian	159	Reference			
Black/African American	17	1.585 (0.675 - 3.725)	0.290		
White	185	1.323 (0.909 - 1.928)	0.144		
AFP(ng/ml)	279		0.773		
≤ 400	215	Reference			
> 400	64	1.075 (0.658 - 1.759)	0.772		
Histologic grade	368		0.762		
G1	55	Reference			
G2	178	1.162 (0.686 - 1.968)	0.577		
G3&G4	135	1.222 (0.710 - 2.103)	0.469		
Pathologic stage	349		<0.001***		
Stage I	173	Reference		Reference	
Stage II	86	1.416 (0.868 - 2.312)	0.164	1.362 (0.833 - 2.229)	0.218
Stage III&IV	90	2.823 (1.862 - 4.281)	<0.001***	2.712 (1.782 - 4.126)	<0.001***
Vascular invasion	317		0.169		
No	208	Reference			
Yes	109	1.344 (0.887 - 2.035)	0.163		
Residual tumor	344		0.203		
R0	326	Reference			
R1&R2	18	1.604 (0.812 - 3.169)	0.174		
TMEM88	373		0.033*		
Low	186	Reference		Reference	
High	187	0.687 (0.485 - 0.971)	0.033*	0.740 (0.510 - 1.072)	0.111

*P<0.05, ***P<0.001.

### TMEM88 was correlated with immune infiltration of HCC

**Figure 5:**
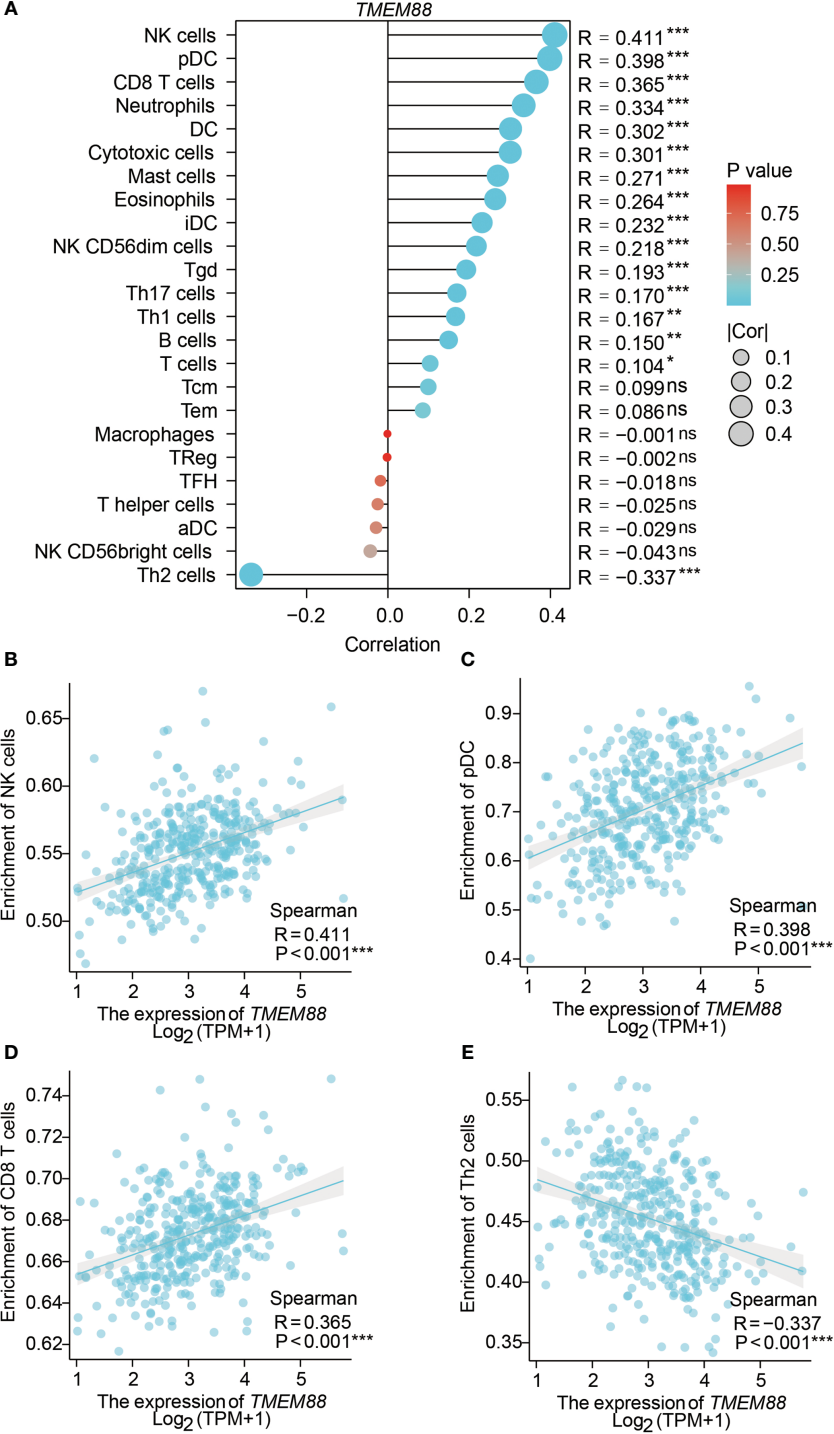
TMEM88 was correlated with immune cell enrichment in HCC microenvironment. **(A)** The correlations between immune cell enrichment and TMEM88-mRNA level in HCC tissues were summarized. Spearman correlation tests indicated positive correlations between TMEM88-mRNA level and NK cell enrichment **(B)**, pDC cell enrichment **(C)**, CD8+ T cell enrichment **(D)**. In contrast, TMEM88 level was negatively correlated with Th2 cell enrichment **(E)**. *P<0.05, **P<0.01,***P<0.001, ns, not significant.

By analyzing the correlations between TMEM88 levels with the immune cell enrichment, we observed that TMEM88 may be involved in the HCC tumor microenvironment (**Figure 5A**). For example, higher TMEM88 was positively correlated with NK cell enrichment (**Figure 5B**), pDC cell enrichment (**Figure 5C**), CD8+ T cell enrichment (**Figure 5D**). In contrast, TMEM88 level was negatively correlated with Th2 cell enrichment (**Figure 5E**).

### TMEM88 suppressed HCC progression both *in vitro* and *in vivo*

**Figure 6:**
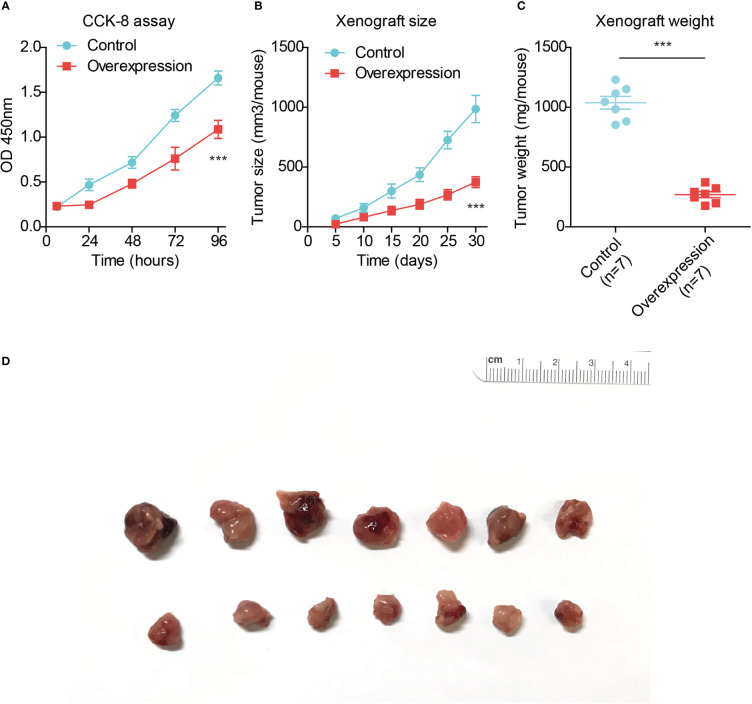
TMEM88 suppressed HCC progression both *in vitro* and *in vivo*. **(A)** CCK-8 assay was conducted to test the proliferation capacity of TMEM88-overexpressed HCC cells and control cells. **(B)** Growth curve of mice subcutaneous xenografts were monitored and recorded. **(C)** The tumor weights of isolated xenografts were tested and compared in the two groups. **(D)** The photographed images of resected xenografts from nude mice. ***P<0.001

Since clinical evidence implied a potential tumor-suppressing role of TMEM88, we next conducted cellular experiments to validate its detailed effects in HCC. TMEM88 plasmids were transfected into Huh7 cells. As a result, CCK-8 proliferation assay revealed an attenuated HCC growth after overexpressing TMEM88 (**Figure 6A**), highlighting the crucial role of TMEM88 as a novel tumor suppressor.

Finally, we conducted *in vivo* analysis using nude mice to establish xenograft model. By monitoring the xenograft growth curve, we confirmed that overexpressing TMEM88 attenuated HCC growth (**Figure 6B**). Consistent with the growth curve, isolated xenografts established by TMEM88-overexpressing cells showed significant lighter tumor weight and smaller tumor size compared to those established by control cells (**Figures 6C, D**).

## Discussion

Involvement of TMEM88 in different malignancies has been reported by different research groups worldwide (15, 16). For example, low TMEM88 expression was identified in both thyroid cancer specimens and corresponding cell lines. Overexpressing TMEM88 remarkably attenuated proliferation and invasion capacities of thyroid carcinoma cells (17). Although a previous gene-sequencing study suggested that TMEM88 combined with several other genes can help distinguish high- or low-risk groups of HCC, their data didn’t illustrated the independent prognostic role of TMEM88 in HCC (18). Meanwhile, their study lacks the experimental verification regarding the detailed roles of TMEM88 in HCC progression.

In this study, we observed a negative correlation between TMEM88 expression and unfavorable prognostic characteristics of HCC, including advanced tumor stage, poorer differentiation grade, and higher serum AFP level. Additionally, we found that higher levels of TMEM88 were associated with improved overall survival and disease-specific survival in HCC patients. We also demonstrated that overexpression of TMEM88 in Huh7 cells resulted in decreased cell proliferation, confirming its role in inhibiting HCC progression. The *in vivo* xenograft experiments further validated the anti-tumor effects of TMEM88 in HCC, which is consistent with previous findings in bladder cancer (19).

Besides tumor progression, TMEM88 was also reported to be associated with drug resistance. Maria et al.’s data showed that platinum resistant xenografts generated by ovarian cancer cells exhibited significantly lower methylation level of TMEM88, comparing to that of platinum sensitive xenografts. Interestingly, although knockdown of TMEM88 enhanced ovarian cancer cell proliferation, it also re-sensitized cells to platinum treatment. Therefore, TMEM88 plays an important role in platinum resistance. Moreover, considering that Wnt signaling is involved in promoting resistance to cisplatin, docetaxel, radiotherapy, etc, it’s high likely that TMEM88 may help restore therapeutic sensitivity in tumors, highlighting its potential role in tumor treatment.

Apart from its role in tumor progression, TMEM88 has also been implicated in drug resistance. For example, in ovarian cancer, lower methylation levels of TMEM88 were observed in platinum-resistant xenografts compared to platinum-sensitive ones. Interestingly, knockdown of TMEM88 in ovarian cancer cells enhanced cell proliferation but also resensitized them to platinum treatment (20), highlighting the potential role of TMEM88 in restoring therapeutic sensitivity in tumors. Furthermore, given the involvement of Wnt signaling in promoting resistance to various therapies (21), including cisplatin, docetaxel, and radiotherapy, TMEM88 may have broader implications in tumor treatment.

Our study has several limitations. Firstly, we didn’t distinguish the subcellular localization of TMEM88 in HCC, which would be an important aspect. Although TMEM88 is normally localized in cell membrane, Zhang’s data and Yu’s data suggested that cytosolic mislocalization of TMEM88 promotes the progression of NSCLC and triple-negative breast cancer, respectively (22, 23). In contrast, nuclear localization of TMEM88 was negatively correlated with lymph node metastasis in triple-negative breast cancer, implying that TMEM88 may play completely distinct tumor-related roles relying on its subcellular localization (23). Secondly, we didn’t dig into the detailed upstream regulators and downstream effectors of TMEM88. It has been reported that miR-708 can directly bind to TMEM88 and inhibits its functions in human hepatic stellate cells (7). Interestingly, overexpression of TMEM88 in lung adenocarcinoma cells also reduced TMEM88 expression and enhanced cancer cell proliferation and invasion (24), suggesting the possible regulating manner in HCC. In addition, promoter methylation of TMEM88 can downregulate TMEM88 expression and participates in modulating its downstream pathways. For example, Ma et al. reported that NSCLC patients with high TMEM88 methylation exhibited worse prognosis than those with low TMEM88 methylation (25). Although the downstream signaling pathway of TMEM88 in HCC was not explored here, it is high likely that TMEM88 functions through inhibiting Wnt/β-catenin signaling pathways as reported in various cancer types (16, 17, 19). Nevertheless, further molecular biological studies will be necessary to further illuminate the signaling network of TMEM88 in different malignancies.

## Conclusions

Taken together, our data suggested an anti-tumor effect of TMEM88 and verified its role as a novel prognostic biomarker in HCC.

## Data availability statement

The original contributions presented in the study are included in the article/supplementary material. Further inquiries can be directed to the corresponding author.

## Ethics statement

The animal study was reviewed and approved by Ethics Committee of Xuzhou Polytechnic College of Bioengineering.

## Author contributions

LC designed this project. YD supervised this study and conducted clinical data analysis. KS conducted cellular experiments. PP helped with mice experiments. FH contributed to statistical analyses. All authors contributed to the article and approved the submitted version.
